# Evaluating changes to home bowel cancer screening kits: an end-user perspective study

**DOI:** 10.1007/s10552-023-01695-x

**Published:** 2023-04-21

**Authors:** L. Myers, M. J. Ireland, B. Viljoen, B. Goodwin

**Affiliations:** 1 Cancer Council Queensland, 553 Gregory Terrace, Fortitude Valley, Brisbane, QLD 4006 Australia; 2 School of Psychology and Well-Being, University of Southern Queensland, Springfield, Australia; 3 School of Nursing and Midwifery, University of Southern Queensland, Toowoomba, Australia; 4 Centre for Health Research, University of Southern Queensland, Springfield, Australia

**Keywords:** Bowel cancer, Screening participation, FOBT, Public health, Cancer screening

## Abstract

**Purpose:**

Many people do not participate in mail-out bowel cancer screening programs due to difficulties using the screening kit. The current study investigated the ways the screening kit could be modified to improve usability.

**Methods:**

1,109 people evaluated 15 different screening kit modifications. Participants reported on how these kit modifications would affect their screening barriers, their future screening intentions, and how much they would recommend that the modification is made to the current screening kit used the program. All responses were given via an online survey conducted between April and December of 2021.

**Results:**

Seventeen percent of previous NBCSP non-participators indicated that a *one-sample* test would increase their intention to participate. Recommendation ratings demonstrated higher levels of support for modifications that included providing a barcode naming label (*M* = 9.06, 95% CI [8.81, 9.31]), having a larger diameter opening of the collection tube (*M* = 8.42, 95% CI [8.10, 8.74]), and highlighting the expiry date on the kit packaging (*M* = 8.59, 95% CI [8.29, 8.89]). There were lower levels of support for modifications that reduced the size of the packaging the kit is sent in (*M* = 6.47, 95% CI [6.09, 6.85]), removed branding from kit packaging (*M* = 5.98, 95% CI [5.57, 6.39]), and removed the information booklet that comes with the screening kit (*M* = 5.25, 95% CI [4.78, 5.72]).

**Conclusion:**

These findings highlight multiple ways in which bowel cancer screening kits can be changed to increase usability for invitees of national bowel cancer screening programs. Findings have implications for all screening programs that use immunochemical-based bowel cancer screening kits.

**Supplementary Information:**

The online version contains supplementary material available at 10.1007/s10552-023-01695-x.

## Introduction

Globally, bowel cancer is the second leading cause of cancer-related death and represents 10% of all cancer cases [1]. As early detection of bowel cancer improves treatment efficacy and can lead to increased survival rates, population bowel cancer screening programs aim to increase the rate of early detection to reduce the burden of the disease [2].

Consequently, many countries have implemented mail-out bowel cancer screening programs [3]. In the Australian National Bowel Cancer Screening Program (hereafter referred to as the NBCSP), people between 50 and 74 years are mailed fecal occult blood tests (FOBT) directly to their home [4]. Invitees are asked to collect two small stool samples from two separate bowel motions using the collection tubes provided. These samples are then to be kept in the zip lock bag provided and stored in the fridge to ensure they are not degraded by heat. Invitees are supplied with a pre-paid envelope to return their samples for processing. If the FOBT is positive, the invitee and their nominated general practitioner (GP) are notified to schedule a colonoscopy for follow-up testing and treatment. This process occurs with little to no out-of-pocket expenses for the invitee [4].

The introduction of the NBCSP has greatly reduced the incidence and mortality rate of bowel cancer in Australia, and similar improved outcomes have been reported from other population bowel cancer screening programs [5, 6]. However, the efficacy of these programs, particularly in Australia, could be significantly improved through higher participation [5]. The current participation rate in the NBCSP is 43% and is as low as 33.5% for invitees aged between 50 and 54 years [4]. If the NBCSP participation rate could be increased to 60%, it is estimated that 83,800 lives could be saved by the year 2040 [5].

A recent study identified a range of common barriers invitees perceive as making it difficult for them to participate in the NBCSP [7]. Upon receiving the FOBT kit, invitees report procrastination and many, without a sufficient plan for where they are going to keep the kit and when they are going to complete it, ultimately forget to do so [8]. People may also be reluctant to participate in the NBCSP because they feel their autonomy regarding their health care is threatened. Invitees may show avoidance to participate for fear of receiving a cancer diagnosis. Physical difficulties in collecting a stool sample may prevent participation for all recipients including those with restricted mobility or dexterity. Finally, levels of disgust in the testing procedure itself may prevent people from completing and returning their testing kit [7].

Among the most consistently effective interventions to help people overcome these barriers and increase participation involved altering the contents of the FOBT kit [9]. For example, by altering from an older guaiac-based kit to a newer immunochemical kit. This meant participants were required to take fewer samples and the need for dietary restrictions was removed; a change that has led to increased participation rates in several programs [10]. Other changes such as including a toilet liner to aid in collecting the sample and providing rubber gloves have also led to increased participation [11]. Even though the Australian NBCSP already distributes two-sample immunochemical kits and provides toilet liners, participation remains low [4]. This might indicate that further modifications of the current NBCSP kit are needed to improve the ease of use and increase participation rates.

The aim of the current study was to evaluate a range of potential modifications that could be made to the current NBCSP screening kit via a large survey panel of target recipients. Evaluations were made using (1) participants’ perceived barriers to participating in home FOBT screening, (2) future screening intentions, and (3) endorsement or recommendation that the modification is made to the current screening kit.

## Methods

### Participant recruitment

Participants were recruited through paid Facebook advertising and through distributing survey links to various community groups (e.g., community centers and volunteer organizations). Participants were offered the chance to win one of three grocery vouchers as incentives (valued between $20 and $50). Participants needed to be between the ages of 50–74 years (i.e., the age of NBCSP invitees), have access to the internet, and be able to read English. Data were collected between the 21st of April 2021 and the 5th of December 2021.

This study was part of a larger research project whereby one survey link was distributed to potential participants directing them to an online survey with multiple components [12, 13]. In total, 8,584 people clicked the link to take part in the online survey. Given this recruitment method, no further details could be collected for those that did not respond to the survey advertisement. Of these 1,839 viewed the first page of the survey and 1,542 consented to take part in the survey. Two participants did not start the survey as they indicated that their age was below 50. In total, 1,109 (72.0% of consenting participants) completed the kit modification section of the survey. Participants that did not return their last mailed FOBT kit were somewhat less likely to complete the survey *χ*^2^(1) = 7.55, *p* < 0.01, Δ = 7.1%. There was no significant variation in survey attrition according to age or gender (see Online Resource 2 for details). As this was an exploratory study, power calculations were not performed. A target was set of 30 people responding to each modification that also reported one (or more) of the five most common barriers to FOBT participation [7]. This was the smallest stratum of clinical importance that was still practically achievable with available resources and participant capacity to view limited modifications before experiencing fatigue.

### Materials

**Fig. 1:**
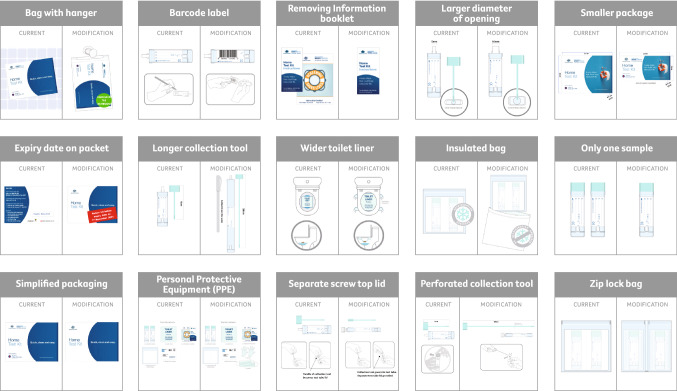
Depiction of FOBT screening kit modifications

The 15 diagrams of kit modifications used in this study are shown in Fig. 1. The current kit used in the NBCSP is depicted on the left-hand side of each panel and the modification to be evaluated by the participant is shown on the right-hand side of each panel. These modifications were based on findings from a previous study [14]. In that study, a human-centric design approach was taken and consultations with ‘end-users’ (i.e., people who receive an NBCSP testing kit) were conducted to identify what aspects of home FOBT screening kits could be modified to increase usability [14].

### Procedure

Participants completed an anonymous online survey delivered on the Qualtrics survey website [15]. The sections relevant to this study took approximately 30–40 min to complete. Prior to seeing the survey, participants provided informed consent and ethical approval for this research was granted by a university-based ethics committee (ref: H19REA291). Participants completed each measure in the order outlined below (also see Online Resource 2).

### Measures

#### Demographics

At the beginning of the survey, participants were asked to report their age (in years) and the gender they identified as. At the end of the survey, participants were asked to provide their country of birth, Aboriginal and Torres Strait Islander status, employment status, and their highest level of education. Residential postcode was used to classify participants by geographic remoteness and socio-economic status according to the Australian Bureau of Statistics, Australian Statistical Geography Standard, and Socio-Economic Indexes for Areas classification system [16, 17].

#### NBCSP history

Participants were asked if they had ever received a bowel cancer screening kit from the NBCSP in the mail before (“yes” or “no”). Those who had were asked if they completed the last FOBT kit that was mailed to them (“yes” or “no”).

#### Future screening intention

Participants were asked “Overall how likely are you to complete and return this home bowel cancer screening kit next time you receive it?” on a scale of 1 (“unlikely”) to 10 (“likely”). To establish the reliability of this measure (needed for the reliable change index described in the data analysis section) a subset of participants (*n* = 330) was asked this item again after completing questions about barriers to bowel cancer screening. The test–retest reliability between these responses was *r* = 0.98, *p* < 0.001.

#### Barriers to home bowel cancer screening (BB-CanS)

Participants responded to the 49 items from the BB-CanS scale [7]. The BB-CanS scale contains items that reflect barriers people may experience while participating in home bowel cancer screening. Participants respond to statements on a 1 (“Not true or would not prevent me from using the test kit at all”) to 4-point (“This would definitely prevent me from using the kit”) scale. There are four subscales in the BB-CanS; disgust (e.g., “It is unhygienic to store a stool sample in my house”), physical difficulty (e.g., “I do not think that I could use the home test kit correctly”), fear or avoidance of bowel cancer screening (e.g., “I’m scared to find out if I have cancer”), and a perceived lack of autonomy (e.g., “my health care is between me and my doctor”). Internal consistency was high in the current sample ranging from Ω = 0.88 for autonomy to Ω = 0.96 for disgust [18]. The BB-CanS also contains three stand-alone items that represent the most commonly reported barriers to FOBT screening participation [7]. These are “My lack of planning means I will never get around to it,” “I will probably put the kit somewhere and forget about it,” and “I do not need to complete a home bowel cancer screening test as I have had a colonoscopy or another test separate from the National Bowel Cancer Screening Program.”

#### Changes to BB-CanS items

After completing the BB-CanS, participants were shown modifications from Fig. 1, along with the accompanying description of the modification (see Online Resource 1). They were then asked, for example, “Please rate the degree to which you believe that *including a separate screw top lid* as depicted above would have an influence on these barriers” on a scale of 1 (“This modification would *not* help reduce this barrier at all”) to 4 (“This modification would remove this barrier all together”). To reduce the response burden, the participant would only be asked about the barriers they previously stated would affect their participation (i.e., responded with a 2 or more in the initial BB-CanS scale). To further reduce the participation burden, participants were only shown a random selection of two or four FOBT screening kit modifications based on the number of barriers they report experiencing with home bowel cancer screening (see Online Resource 2).

#### Change in intentions

After each presentation of an FOBT kit modification, participants were asked “Overall, how likely would you be to complete and return your home bowel cancer screening kit next time you receive it *if it included this modification*?” on a scale of 1 (“unlikely”) to 10 (“likely”).

#### Recommendation of kit modification

After each presentation of an FOBT kit modification, participants were also asked “To what degree *do you recommend including this modification* in the current National Bowel Cancer Screening Program?” on a scale of 1 (“definitely do not recommend”) to 10 (“highly recommend”).

#### Further comments

After being shown an FOBT kit modification, participants were asked “Do you have any comments or suggestions regarding this modification?” with an open-text box to provide a written response.

### Data analysis

All analysis was done in R within the R studio environment [19, 20]. Given the exploratory nature of the study, with no specific hypotheses to test, a descriptive-analytic approach was taken [21]. All available data were used in the analysis and no methods of imputation were used.

#### Changes to BB-CanS items

**Fig. 2:**
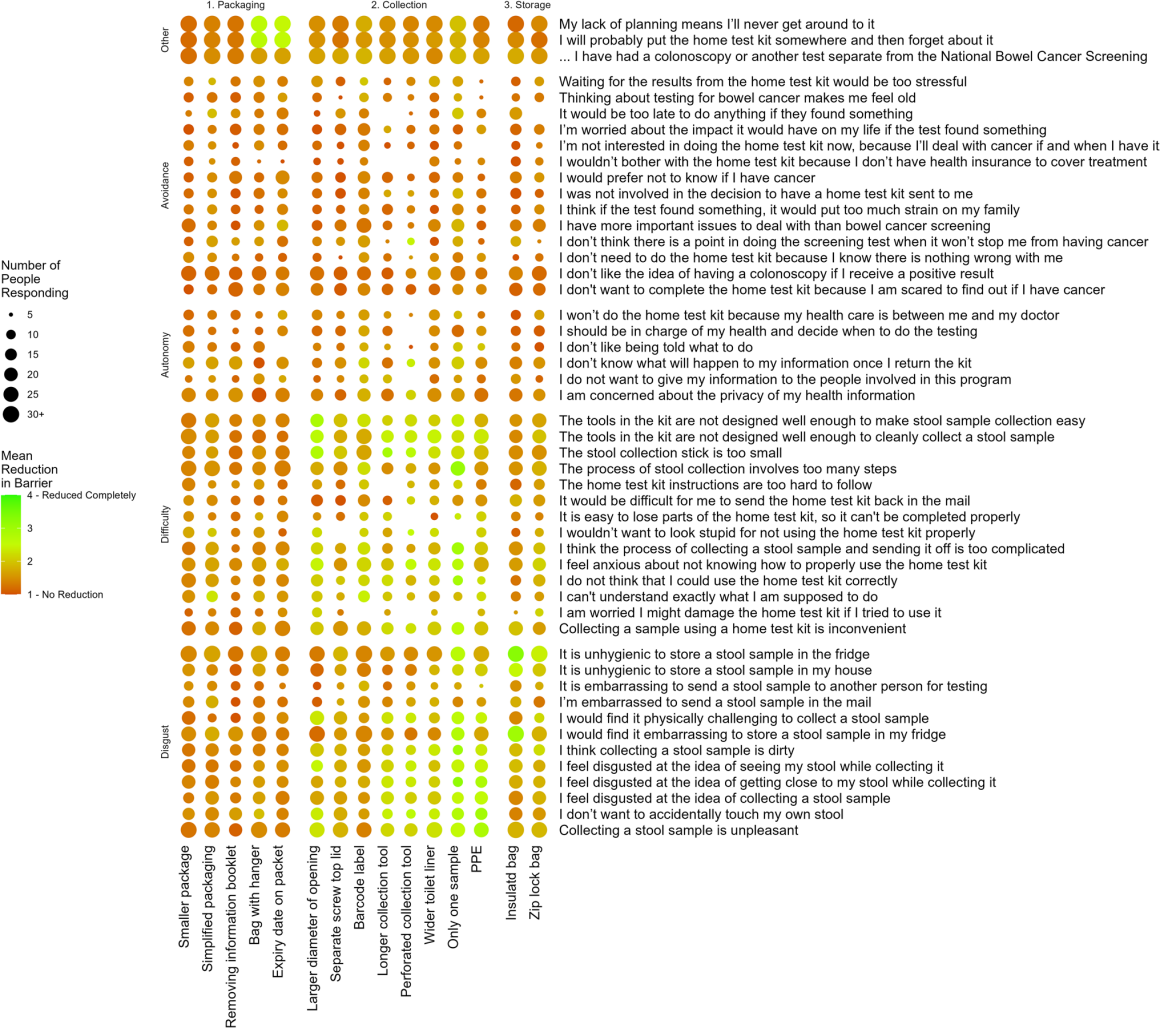
Mean change in BB-CaNs items

The average response was calculated for each *Change to BB-CanS item* for each modification. These averages were plotted using a balloon plot (see Fig. 2), whereby the color of each ‘balloon’ represents the mean value and the size of each balloon represents the number of responses given.

#### Future screening intention

A reliable change index (RCI) approach was used to assess change in intention status [22]. An RCI indicates if a person’s change in response is above and beyond what would be expected given standard error of measurement of the measure (see [22]). For example, if a person’s change was measured as a two but the standard error of measurement for that item is three, then it is unclear if that change was due to an actual change in the participant or an artifact of measurement error. An RCI takes this measurement error into account and indicates if a person’s change in an outcome is beyond what could be expected given the error in the measurement and thus provide stronger evidence that a real change has occurred.

Participants were categorized as having a reliable increase in intention level, reliable decrease in intention level, or as having no reliable change in intention level [22]. The number of people falling into these categories was further compared between those who completed and returned their last NBCSP kit and those that did not.

#### Recommendation levels

The percentage of participants giving each recommendation rating was calculated and displayed in a histogram for each kit modification.

#### Text comments

Comments for each FOBT kit modification were analyzed using content analysis [23]. Codes were based on common themes from the comments left for each FOBT kit modification. A codebook that lists each code with its definition was used to code each comment (see Online Resource 3). Two independent coders coded all comments. Both the percentage agreement between coders and Kappa interrater reliability statistic were calculated using the irr package in R [24]. All disagreements were resolved through discussion. The frequency and percent (i.e., the frequency of each code divided by the frequency of all codes for each modification) of each code’s occurrence were calculated.

## Results

A total of 1,109 people provided data evaluating the FOBT kit modifications with a mean age of 61.75 years (*SD* = 6.92); the remaining demographic statistics are provided in Table 1. In this sample, 339 (32.98%) did not complete and return their last NBCSP FOBT kit. To ensure responses were representative of those for whom kit modification interventions would apply, participants who indicated that they have not taken part in the NBCSP because either (a) according to the instructions sent with the kit indicating they were not required to complete the test, (b) their GP advised them against completing the kit, or (c) they have recently completed a colonoscopy were considered ineligible for the NBCSP and not included in the analysis of this study.

**Table 1:** Demographic statistics

Demographic		*n*	(%*)
Gender			
Male		435	(39.30)
Female		668	(60.34)
Did not report		4	(0.36)
Born in Australia			
Yes		734	(72.53)
No		278	(27.47)
ABTSI			
Yes		17	(1.68)
No		982	(97.13)
Not disclosed		12	(1.19)
Education			
University		499	(50.76)
TAFE/Apprenticeship		240	(24.42)
High school or lower		244	(24.82)
SEIFA			
1st quintile (most disadvantaged)		126	(11.51)
2nd		165	(15.07)
3rd		198	(18.08)
4th		335	(30.59)
5th quintile (least disadvantaged)		271	(24.75)
ARIA			
Major city		691	(63.11)
Inner regional		259	(23.65)
Outer regional		130	(11.87)
Remote		10	(0.91)
Very remote		5	(0.46)

*ABTSI* Aboriginal and Torres Strait Islander, *SEIFA* Socioeconomic index for areas, *TAFE* Technical and Further Education, *ARIA* Accessibility and Remoteness Index of Australia

*Valid percent

### Kit modifications and changes in barriers to bowel cancer screening

The mean change in barriers associated with each kit modification is shown in Fig. 2. To aid in interpretation, reduction in barriers were grouped according to the subscales of the BB-CanS [7] and the kit modification was grouped according to whether they altered the packaging, the collection process, or the storage of the FOBT kit. Overall, the highest mean reduction in BB-CanS barriers was typically recorded for FOBT kit modifications that altered the *collection process*, such as requiring only one sample to be collected and providing PPE. These reductions were mainly evident for items in the disgust and difficulty BB-CanS subscales. While making modifications to the FOBT kit *packaging* did not have a noticeable effect across items in the BB-CanS subscales, modifying the FOBT kit *packaging* by providing a *bag with a hanger* and highlighting the *expiry date on the packet* tended to reduce barriers relating to “My lack of planning means I will never get around to it” and “I will probably put the kit somewhere and forget about it.” Modifications to how people can store their FOBT kit between and after the collection stages resulted in a higher reduction in barriers, such as “It is unhygienic to store a stool sample in the fridge” and “I would find it embarrassing to store a stool sample in my fridge,” but with smaller to no effects across the remaining barriers.

### Future screening intention

**Fig. 3:**
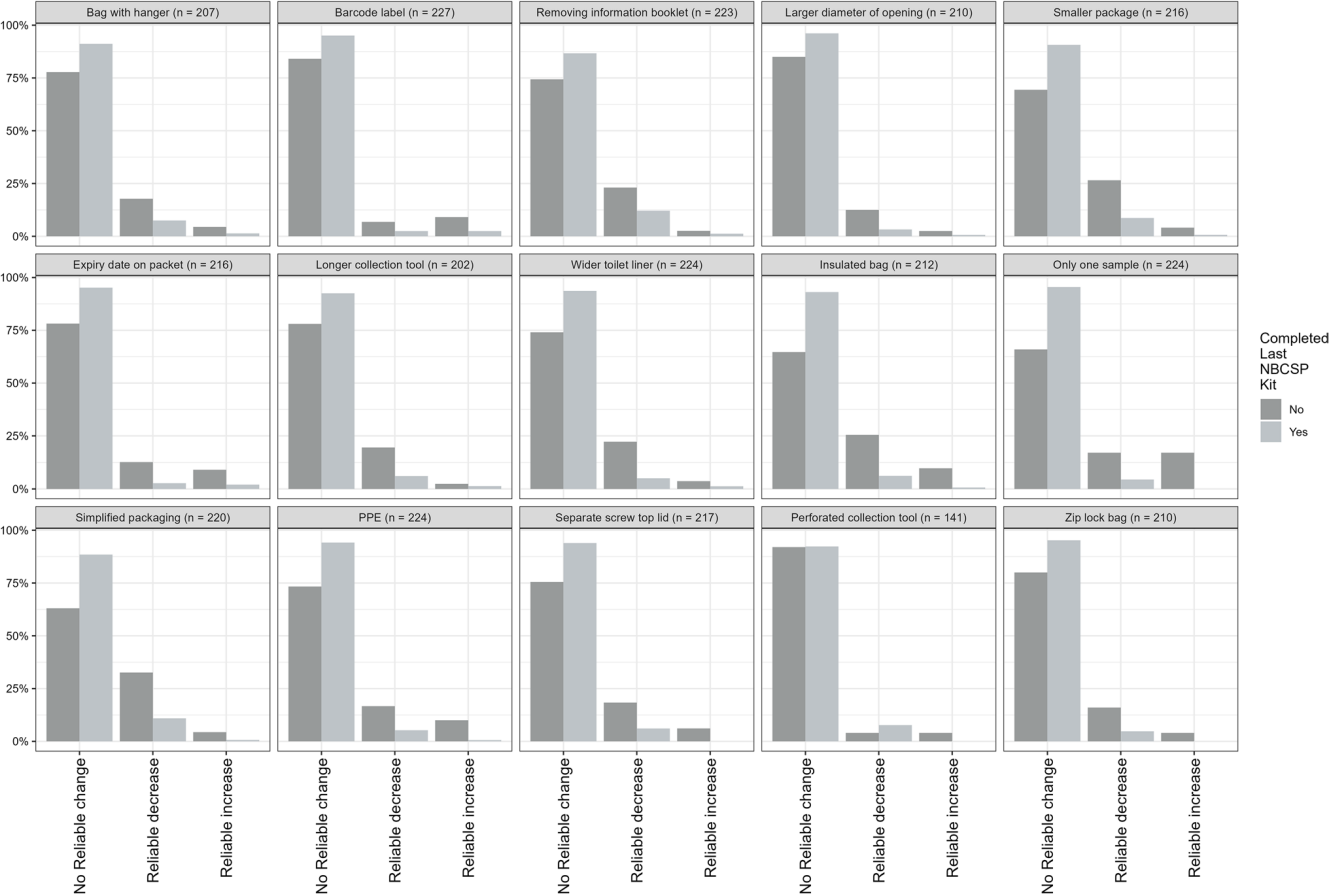
Percentage of reliable change in screening intention

The percentage of reliable change associated with each FOBT kit modification is presented in Fig. 3. For the vast majority of people, no reliable change in screening intention occurred in response to each kit modification. Those that did not return their last NBCSP kit did tend to show a higher percentage of people with a reliable change in intention levels and this was most often a reliable decrease in screening intention. The kit modifications that resulted in the highest percent of people indicating a reliable *increase* in screening intention was requiring *only one sample* for previous NBCSP non-participators (17.02%) and including an identification barcode label for previous NBCSP participators (2.45%). The kit modifications that resulted in the highest percentage of people indicating a reliable *decrease* in screening intention were *simplified packaging* for previous NBCSP non-participators (32.60%) and *removing the information booklet* for previous NBCSP participators (12.12%).

### Recommendations

**Fig. 4:**
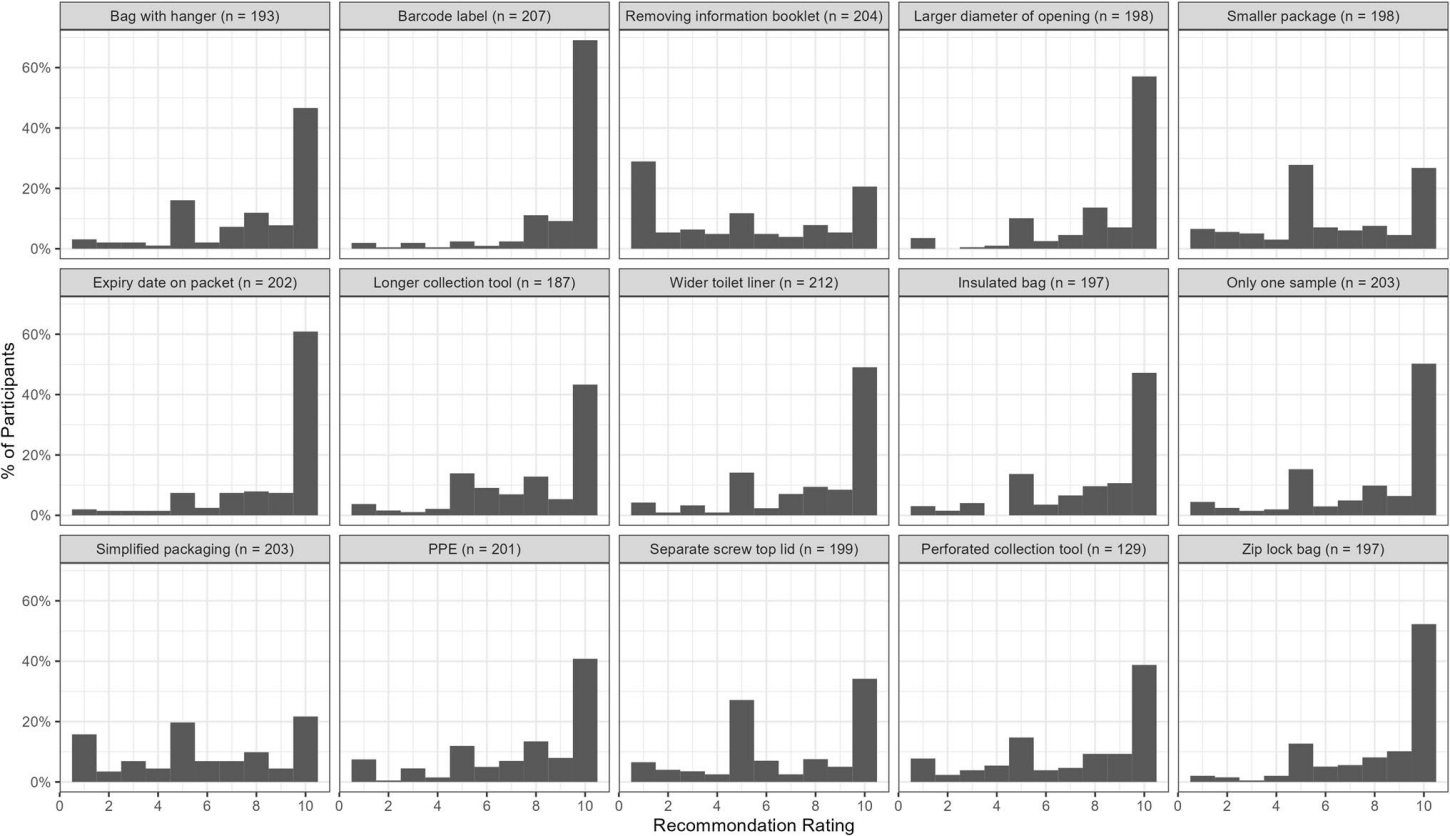
Recommendation ratings for modifications

The recommendation ratings for each FOBT kit modification can be found in Fig. 4. All kit modifications had ratings that skewed toward higher levels of recommendation, except for the *removing information booklet, smaller packaging*, and *simplified package* modifications; these had flatter or multimodal distributions of recommendation ratings. The modification of including an *identification barcode label* had the highest recommendation ratings and the modification of *removing the information booklet* had the highest percentage of low recommendation ratings.

### Text analysis

The results of the content analysis are shown in Table 2. There was “substantial” interrater agreement for all the codes, with 86% agreement between the coders, equating to a Cohen’s Kappa of 0.806 [25]. There was a mix of positive and negative sentiments toward the kit modifications. There were generally positive sentiments for modifications such as, including an *identification barcode label*, having a *larger diameter of the opening* and highlighting the *expiry date on the packet*, where the modification would assist in the FOBT screening process by making it easier (e.g., *barcode label*: “Brilliant idea. The current surface is challenging to write on”) or more sanitary (e.g., the *larger diameter of the opening*, “The mod[*sic*] lessens concern over touching stool”). However, for some modifications, such as requiring *only one sample*, having a *separate screw top lid*, and having a *perforated collection tool*, had both positive sentiments in the comments, suggesting the modification would either make the screening process easier and more reliable in one way (e.g., *only one sample*, “This modification would make the process much simpler and convenient”) but raise concerns that it would make the screening process harder and less reliable in other ways (e.g., *only one sample*, “Would rather 2 samples for my own peace of mind”).

**Table 2:** Content analysis of text responses for each kit modification

Modification	Code	%	(*n*)
Bag with hanger	Help prevent losing/procrastinating/forgetting	28.57	(16)
	Concerns about added costs/waste	21.43	(12)
	The modification is unnecessary/won’t affect participation	21.43	(12)
	It would be embarrassing to display	12.50	(7)
	It would be more convenient	7.14	(4)
	May not work for all toilets	5.36	(3)
	Other	3.57	(2)
Barcode label	Easier to use for themselves and those with mobility issues	86.36	(38)
	More reliable and secure	11.36	(5)
	Other	2.27	(1)
Expiry date on packet	Increase awareness of the expiry date	47.83	(11)
	Prompts participation	30.43	(7)
	The modification is unnecessary/won’t affect participation	13.04	(3)
	Might promote delays in participation	8.70	(2)
Insulated bag	The modification is unnecessary/won’t affect participation	37.50	(15)
	Better than storing in the fridge/near food	35.00	(14)
	Concerns over added costs/waste	12.50	(5)
	It would prevent overheating	12.50	(5)
	Other	2.50	(1)
Larger diameter of opening	It would be easier to use for themselves and those with vision impairment	59.18	(29)
	The modification is unnecessary/won’t affect participation	40.82	(20)
Longer collection tool	The modification is unnecessary/won’t affect participation	33.96	(18)
	Easier and more sanitary collection	32.08	(17)
	Longer stick may be harder to use/fragile	28.30	(15)
	Other	5.66	(3)
Only one sample	Concerned one sample is not reliable enough	58.44	(45)
	Easier/less stressful to organize one sample	23.38	(18)
	Removed storage issues	10.39	(8)
	The modification is unnecessary/won’t affect participation	7.79	(6)
Perforated collection tool	The modification is unnecessary/won’t affect participation	28.89	(13)
	It would be harder to use/parts might break or get lost	22.22	(10)
	It would be easier to use	15.56	(7)
	It makes it too complicated	11.11	(5)
	Concerns about added costs/waste	8.89	(4)
	More sanitary collection	8.89	(4)
	Other	4.44	(2)
PPE	It may help those with sanitary concerns	24.36	(19)
	The modification is unnecessary/won’t affect participation	17.95	(14)
	Concerns about added costs/waste	15.38	(12)
	Gloves would be beneficial	15.38	(12)
	Masks would not be helpful	14.10	(11)
	May help the collection process	10.26	(8)
	Concerns about the sizing of the gloves	2.56	(2)
Removing information booklet	Would rather the additional information and instructions	75.86	(44)
	The modification is unnecessary/won’t affect participation	12.07	(7)
	Reduces information burden	10.34	(6)
	Reduced Waste	1.72	(1)
Separate screw top lid	The modification is unnecessary/won’t affect participation	48.08	(25)
	Too complicated and easy to lose parts	25.00	(13)
	Modification looks harder to use	17.31	(9)
	May help those with sanitary concerns	5.77	(3)
	Other	3.85	(2)
Simplified packaging	The modification is unnecessary/won’t affect participation	43.86	(25)
	Might hide the importance of the package	33.33	(19)
	Keeps the purpose of the package more private	7.02	(4)
	Makes screening seem less important	7.02	(4)
	Easier to understand and less confronting	5.26	(3)
	Other	3.51	(2)
Smaller package	The modification is unnecessary/won’t affect participation	47.17	(25)
	This would make it less visible, easier to be forgotten, misplaced, or overlooked	26.42	(14)
	It would reduce waste/costs	11.32	(6)
	It would be more discrete	5.66	(3)
	Other	5.66	(3)
	It would make for easier postage	3.77	(2)
Wider toilet liner	Easier collection and less risk of water contamination	62.50	(25)
	The modification is unnecessary/won’t affect participation	15.00	(6)
	Concerned it would be harder to dispose of	12.50	(5)
	Concerned it brings the stool too close to the body	10.00	(4)
Ziplock bag	The modification is unnecessary/won’t affect participation	64.29	(18)
	It would have better concealment for storage	17.86	(5)
	Other	17.86	(5)

Common themes did occur across all or most FOBT kit modifications. Often participants were concerned about the cost and environmental impacts of adding things to the FOBT screening kit (e.g., *PPE*, “Adds to the cost. I believe washing ones [sic] hands is preferable to tossing possible contamination into land fill”). Participants also noted when the suggested modification would not affect, or was not relevant to, their screening behavior (e.g., *Expiry date on packet*, “Would not make a difference to me”).

## Discussion

This consultation study highlighted multiple ways in which the process of home bowel cancer screening can be improved for screening invitees and provides specific insights into how the FOBT kit currently distributed in the Australian NBCSP can be modified to possibly improve usability. There were mixed findings for the 15 modifications that were evaluated by end-users, with some modifications having strong support, while other modifications have lower levels of support.

Overall, the *barcode label*, *larger diameter of opening*, *expiry date on packet*, *wider toilet liner*, and *providing PPE* (specifically the provision of disposable gloves) modifications had consistently favorable evaluations across the outcome measures. Fortunately, all these modifications, except for making a *larger diameter opening*, do not require changing the FOBT kit itself, rather they are changes to the auxiliary materials sent with the FOBT kit. Therefore, if these modifications were applied, no alteration would be needed in the processing methods of the pathology lab, but the overall usability of the FOBT kit could still be enhanced. It is important that any intervention that increases FOBT screening participation remains cost-effective [26]. The next step in the development of a new FOBT screening kit will need to consider how much making modifications such as these will increase costs to the screening program and weigh that against the increase in participation. Modeling of Australian data has shown that any FOBT screening promotion strategy that can increase participation to 60% will be highly cost-effective if it costs less than $72 million per annum [26].

Some modifications, such as the *longer collection tool* and the *perforated collection tool* seemed to reduce specific barriers to FOBT screening but had lower overall recommendation ratings and received criticism in the text responses. This suggests that while some modifications might reduce some specific screening barriers, they can simultaneously cause other problems in the screening procedure. For example, people reported that the longer stick would make the collection procedure more sanitary but at the same time, it would require more dexterity to maneuver the longer tool into the collection container. Due to the large number of people invited to the national screening program (e.g., over five million each year in Australia) [4], unintended consequences following changes to public health policy and public health interventions can have extensive and detrimental effects and therefore need to be data driven and thoroughly considered [27]. Involving stakeholders in the development of new interventions, such as the end-user evaluation presented in this study, is a key strategy to identify possible unintended consequences before interventions are implemented for the general public [27].

There was very little indication that any of these modifications would increase people’s perceived intention to participate in FOBT screening. Models of health behavior, such as the Health Action Process Approach (HAPA), suggest that one’s *intention* to engage in a health behavior is formed by motivational factors (such as their perceived risk of developing a disease) thoughts regarding positive outcomes occurring if they engage in that health behavior and their confidence in their ability to perform the health behavior [28]. In the current context, the HAPA model would imply that individuals will only have a higher intention to participate in FOBT screening if they believed they were at risk of developing bowel cancer, thought that completing the FOBT kit would lower their risk of dying from bowel cancer, and were confident in their ability to complete FOBT kit [29]. In this case, FOBT kit modifications (e.g., *larger diameter of opening*, *wider toilet liner*, and *PPE*) could improve the volitional factors of FOBT screening (i.e., making the physical actions of FOBT screening easier), but are not likely to affect motivational factors that influence participants screening intention. While these modifications may not increase screening intentions, they may promote FOBT screening participation by helping people during the ‘transition’ phase, where people convert high screening intention into actualised screening participation [28]. As 61% of people that *do not* return their NBCSP kit report having intentions to do so [30], a large majority of non-screeners would benefit from health interventions that facilitate their transition from mere intention to kit completion. Currently, only one reminder letter is issued to those who have not returned their FOBT kit [31]. Future research should investigate all aspects of the invitation process, as well as the instructions sent with the kit, to assess which strategies are needed to help recipients through this transitional phase. For instance, multiple reminders may need to be issued or instructions should be given to keep the kit near the bathroom to prompt participation. Nevertheless, a concerning number of people did indicate a lower level of screening intention regarding some of the FOBT kit modifications; perhaps as a function of lowered confidence in their ability to complete the kit. As such, before any change to the current FOBT screening kit is made, it needs to be demonstrated that such changes will not reduce screening intention and therefore reduce screening participation.

### Strengths and limitations

This study is the first to provide end-user evaluations from a large sample on a wide range of possible modifications to the FOBT kit used in the Australian NBCSP. A comprehensive range of outcome measures, including the validated list of home bowel cancer screening barriers (i.e., the BB-CaNS), further strengthens the conclusions of this study. This was also the first study to use graphical depictions of bowel cancer screening kit modifications based on the specific kit characteristics relevant to the participants. Many countries now use immunochemical-based kits, and while the specific kit may be different, the principles of these findings are likely to have implications for those programs as well. For instance, findings such as highlighting the expiry date or including a barcode label to improve usability would apply regardless of the specific screening kit that is used.

Some limitations need to be considered when interpreting these results. Given that participation in the survey was voluntary, self-selection may bias the result. There were also slight over-representations of females and people that returned their last FOBT kit. Given the large number of modifications that were evaluated, and the large number of outcomes used in this study, it was not feasible to test which modification had statistically significantly higher evaluations nor which combinations of modifications could be trialed together. Future studies might benefit from combining a select few of these FOBT kit modifications and directly test preferences in reference to the current screening kit in use. This research also focused on the general population and specific research should be conducted to facilitate participation in vulnerable groups, such as first nations’ people or those with physical impairments. Finally, due to an error in the data collection process, fewer participants evaluated the *Perforated collection tool* modification. However, asides from the smaller sample size for this kit modification, this is unlikely to bias the result.

## Conclusion

The burden of bowel cancer can be greatly reduced through greater participation rates in national screening programs. This study provides vital information about the ways in which the FOBT kit can be changed to minimize barriers experienced by those willing to engage in bowel cancer screening. It is unlikely that any one of these modifications used in isolation will dramatically increase participation rates. However, these findings can be used to inform improvements to FOBT kits that may involve multiple modifications. It is vital that evidence from end-users continues to inform how these programs can be adapted to improve usability. These findings have direct implications for the Australian NBCSP and can inform other national mail-out FOBT screening programs.

## Supplementary Information

Below is the link to the electronic supplementary material.

Supplementary file1 (DOCX 15 KB)

Supplementary file2 (DOCX 61 KB)

Supplementary file3 (DOCX 20 KB)

## Data Availability

All (de-identified) data, materials, and code for this manuscript are available upon reasonable request.
