# Supplementary material for: Evaluating changes to home bowel cancer screening kits: an end-user perspective study
Source: Cancer Causes Control. 2023 Apr 21;34(7):583–94. doi: 10.1007/s10552-023-01695-x (PMC10202976; doi:10.1007/s10552-023-01695-x)
Supplement: Supplementary file 1 — Supplementary file1 (DOCX 15 KB) [file 10552_2023_1695_MOESM1_ESM.docx]

**Online Resource 1**

*Description of Screening Kit Modifications*

| **Name** | **Part that is Changed** | **Description given to participants** |
| --- | --- | --- |
| Bag with hanger | Change to packaging | Currently, the National Bowel Cancer Screening Program screening test kit is packaged in a cardboard envelope and does not include a prompt to store the kit in the bathroom or within reach of the toilet. In this modification, the cardboard envelope comes in a waterproof sealed bag with a hook so that it can be hung on a doorknob or hook. The bag has a sticker on it reminding the user to hang the kit in the bathroom. |
| Barcode label | Change to collection | The label on the tube for the current kit must be filled in by the user using a pen. In the modified version, an identification barcode sticker is provided for the user to place on the test tube so that the user does not have to fill out a label. |
| Removing information booklet | Change to packaging | Currently, the National Bowel Cancer Screening Program kit includes an instruction pamphlet and an information booklet that details facts about bowel cancer including risks and symptoms. In the modification, the information booklet is not included. |
| Larger diameter of opening | Change to collection | The diameter of the opening to the test tube in the current kit is 5 mm wide. The user must insert the collection tool with the sample into this opening. In the modified version, the diameter of the opening is 10mm |
| Smaller package | Change to packaging | The current packaging for the National Bowel Cancer Screening Program kit is larger than the size of a regular envelope. In the modification, the envelope is the size of a regular letter. Both envelopes are made out of thin cardboard. |
| Expiry date on packet | Change to packaging | Currently, an expiry date is printed on the back of the National Bowel Cancer Screening Program screening test kit package. In this modification, there is a bright label on the front of the packaging advising you to return your kit by the expiry date. |
| Longer collection tool | Change to collection | The collection tool in the kit is used to scrape over the surface of the stool to collect a sample. The current collection tool is 6 centimetres long. In the modification, the collection tool is three times the length at 18cm long. |
| Wider toilet liner | Change to collection | The current National Bowel Cancer Screening Program kit includes a flush-able paper liner that sits on the water in the toilet bowl. This is to ensure that the user's stool does not come into contact with the water and to help the user collect their sample. In the modification, this toilet liner is wider and can be attached to the toilet seat under the lid. |
| Insulated bag | Change to storage | Currently, users of the National Bowel Cancer Screening Program are required to store their stool samples in the refrigerator until they are ready to be mailed back. The modification features a zip-locked insulated bag in which the stool samples can be stored so that users do not have to store samples in the refrigerator. |
| Only one sample | Change to collection | Currently, the National Bowel Cancer Screening Program test kit requires the user to collect two samples from two different bowel movements. In the modification below, only one stool sample from one bowel movement is required. |
| Simplified packaging | Change to packaging | The packaging for the current National Bowel Cancer Screening Program kit includes references to bowel cancer and pathology services. The modification features packaging where all reference bowel cancer and pathology services have been removed. |
| Personal Protective Equipment (PPE) | Change to collection | Currently, the National Bowel Cancer Screening Program test kit does not include any personal protective equipment such as gloves or a face mask. In the modification, the kit includes two sets of gloves and two face masks. |
| Separate screw top lid | Change to collection | The current collection tool is inserted into the tube and the handle clips in and becomes the lid of the test tube. In the modified version the collection tool goes into the test tube and a separate screw top lid is provided for the test tube. |
| Perforated collection tool | Change to collection | The current collection tool is 6 centimetres long. Once the sample has been collected, the user places the collection tool into the test tube and twists the handle so it fits onto the tube as a lid. In the modification, the collection tool is 18 centimetres long and perforated so that the user can insert the tool into the test tube and break it off then screw on a separate lid to seal the tube. |
| Ziplock bag | Change to Storage | Currently, the National Bowel Cancer Screening Program test kit provides a padded bag with separate compartments to store each sample in. This padded bag goes into a zip lock bag for storage in the fridge. This means that you need to re-open the zip lock bag to place the second sample inside. In the modification, the zip lock bag has two separate compartments that can be opened and closed separately. |
