# Supplementary material for: Evaluating changes to home bowel cancer screening kits: an end-user perspective study
Source: Cancer Causes Control. 2023 Apr 21;34(7):583–94. doi: 10.1007/s10552-023-01695-x (PMC10202976; doi:10.1007/s10552-023-01695-x)
Supplement: Supplementary file 2 — Supplementary file2 (DOCX 61 KB) [file 10552_2023_1695_MOESM2_ESM.docx]

**ESM 2: Participant Flow Chart**


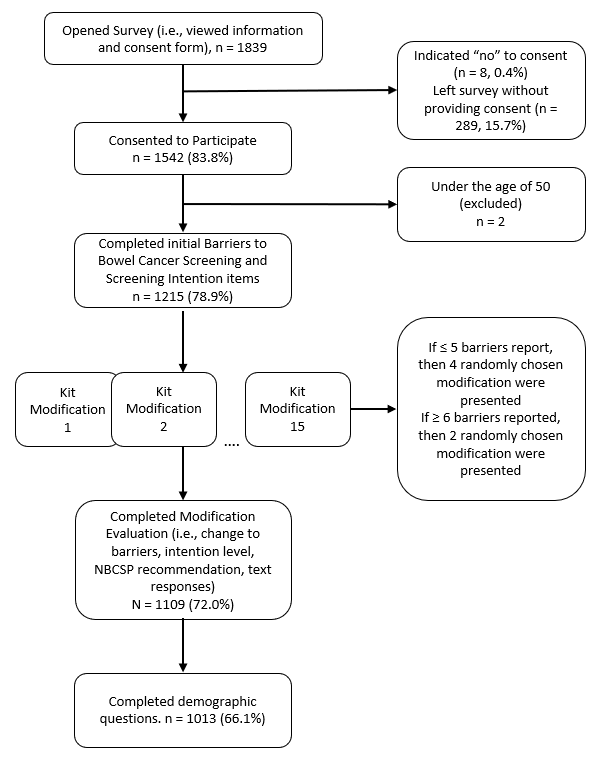


Supplementary Table 1. Consenting, eligible participants’ (n= 1540) attrition according to gender and kit return

|  | Left before completing part 1 | Left before completing part 2 | Left before completing part 3 | Finished survey |
| --- | --- | --- | --- | --- |
| **Gender** |  |  |  |  |
| Male n (%) | 135 (22.4 %)* | 168 (27.9%) | 200 (33.2%) | 403 (40.0%) |
| Female n (%) | 142 (16.2%) | 209 (23.8%) | 273 (31.1%) | 604 (68.9%) |
| **Returned kit** |  |  |  |  |
| No n (%) | 72 (15.9%)* | 113 (25.0%)* | 141 (31.2%)* | 311 (68.8%)* |
| Yes n (%) | 95 (11.4%) | 141 (17.0%) | 200 (24.1%) | 630 (75.9%) |

*Note: values in table are based on participants who provided gender and/or kit return data. Some participants left the survey before providing this information.*

**Chi square test showed significantly more likely to have dropped out by this time point (p <.05)*

Point biserial correlations showed that age was not associated with leaving the survey before part one (*r* = -.01, *p* =.81), two (*r* = -.02, *p* =.43), or three (*r* = -.00, *p* = .90).
