# Supplementary material for: Evaluating changes to home bowel cancer screening kits: an end-user perspective study
Source: Cancer Causes Control. 2023 Apr 21;34(7):583–94. doi: 10.1007/s10552-023-01695-x (PMC10202976; doi:10.1007/s10552-023-01695-x)
Supplement: Supplementary file 3 — Supplementary file3 (DOCX 20 KB) [file 10552_2023_1695_MOESM3_ESM.docx]

ESM 3 – Content Analysis Code Book

| **Bag with hanger** | |
| --- | --- |
| Help prevent losing/procrastinating/forgetting | Comments noting that the modification would help those not forget, lose, or procrastinate completing the FOBT kit |
| It would be more convenient | Comments regarding the modification making it more convenient to use the kit |
| General | Statements that are not specific to the modification |
| May not work for all toilets | Concerns about the modification not working in all toilets |
| Concerns about added costs/waste | Comments or concerns regarding the modification increasing waste or costs |
| The modification is unnecessary/won't affect participation | Comments regarding the modification being unnecessary or not being related to their FOBT participation (or non-participation) |
| Other | Comments that are one-off and do not fit within reoccurring themes |
| It would be embarrassing to display | Comments regarding the prominent display of the FOBT kit being embarrassing or being uncomfortable with it |
| **Barcode label** | |
| Easier to use for themselves and those with mobility issues | The barcode system would be easier to use than the current system. This can be for themselves or those with mobility issues. |
| Other | Comments that are one-off and do not fit within reoccurring themes |
| More reliable and secure | The barcode system would be less prone to errors and/or keep their identity secure |
| **Removing information booklet** | |
| Would rather the additional information and instructions | The modification is not beneficial as they would prefer to have more information than less information (this relates to both bowel cancer information as well as the instructions on how to complete the FOBT kit) |
| Reduces information burden | The modification is beneficial as there is less information to process upon kit receipt |
| Not relevant to participation | The modification is not related to their FOBT participation |
| Reduced Waste | The modification would result in less waste |
| **Larger diameter of opening** | |
| Easier to use for themselves and those with vision or mobility issues | The larger diameter hole would be making the sampling procedure easier. This can be for themselves or others with vision/mobility issues |
| Unnecessary/not related to participation | Comments regarding the modification being unnecessary or not being related to their FOBT participation (or non-participation) |
| **Smaller package** | |
| Easier postage | Comments stating that the smaller package would make postage easier |
| Less visible, easier to be forgotten, misplaced, or overlooked | Comments stating that the smaller package would easier to lose/forgotten about or overlooked among the rest of the mail |
| It would be more discrete | Comments stating that the smaller package would more discrete, and the contents of the package would be less obvious to others when returning the sample |
| Unnecessary/not related to participation | Comments regarding the modification being unnecessary or not being related to their FOBT participation (or non-participation) |
| It would reduce waste/costs | Comments stating that the smaller package would potentially lead to lower costs and less waste |
| **Expiry date on packet** | |
| Increase awareness of the expiry date | Comments suggesting the modification will increase awareness that the FOBT kit has an expiry date |
| Might promote delays in participation | Comments that suggest the highlighted expiry date may promote procrastination |
| The modification is unnecessary/won't affect participation | not related to their FOBT participation (or non-participation) |
| Prompts participation | Comments that suggest the highlighted expiry date may prompt participation |
| **Longer collection tool** | |
| Easier and more sanitary collection | Comments suggesting the longer stick will be easier to use and/or be more hygienic |
| The longer stick may be harder to use/fragile | Concerns that the length of the stick would make it harder to use and/or easier to break |
| The modification is unnecessary/won't affect participation | Comments regarding the modification being unnecessary or not being related to their FOBT participation (or non-participation) |
| **Wider toilet liner** | |
| Concerned it brings the stool too close to the body | Concerns that this modification would bring the stool closer to the body during sampling |
| Easier collection and less risk of water contamination | Comments suggesting that the modification would make the collection easier and/or help reduce the risk of water contaminating the sample |
| Concerned it would be harder to dispose of | Concerns that the modification would not be as easy to dispose of |
| The modification is unnecessary/won't affect participation | Comments regarding the modification being not being related to their FOBT participation (or non-participation) |
| **Insulated bag** | |
| Better than storing in the fridge/near food | Comments about how modification could help with concerns regarding storing the FOBT sample in the fridge/near food |
| Concerns over added costs/waste | Concerns that the modification would result in high costs and/or added waste |
| It would prevent overheating | Comments suggesting that the cooler bag would help prevent the sample from overheating |
| The modification is unnecessary/won't affect participation | Comments regarding the modification being unnecessary or not being related to their FOBT participation (or non-participation) |
| **Only one sample** | |
| Concerned one sample is not reliable enough | Concerns that only one sample would not be as accurate/reliable than collect two samples |
| Easier/less stressful to organise one sample | Comments suggesting that one sample is easier to collect and less stressful than organising two samples on consecutive days |
| Removed storage issues | Comments suggesting only needing to collect one sample would alleviate problems related to storing samples |
| The modification is unnecessary/won't affect participation | Comments regarding the modification being unnecessary or not being related to their FOBT participation (or non-participation) |
| **Simplified packaging** | |
| Easier to understand and less confronting | Comments suggesting that the modified packaging would be easier to understand and be less confronting |
| Keeps the purpose of the package more private | Comments suggesting that the modified packaging helps keep the contents of the package more private |
| Makes screening seem less important | Concerns that the simplified packaging may reduce the importance of bowel cancer screening |
| Might hide the importance of the package | Concerns that the simplified packaging hides the importance of the package |
| The modification is unnecessary/won't affect participation | Comments regarding the modification being unnecessary or not being related to their FOBT participation (or non-participation) |
| **Personal Protective Equipment (PPE)** | |
| Concerns about added costs/waste | Concerns that the PPE will result in added costs and/or added waste |
| Concerns about the sizing of gloves | Concerns that the gloves provided would not fit everyone’s hands |
| Gloves would be beneficial | Comments stating that the gloves specifically would be beneficial |
| It may help those with sanitary concerns | Comments suggesting that this modification would help those how to have sanitary concerns |
| Masks would not be helpful | Comments specifically stating that the masks would not be helpful/needed |
| It May help the collection process | Comments suggesting that the PPE would help the collection process |
| The modification is unnecessary/won't affect participation | Comments regarding the modification being unnecessary or not being related to their FOBT participation (or non-participation) |
| **Separate screw top lid** | |
| May help those with sanitary concerns | Comments suggesting that the modification would help those with sanitary concerns |
| Modification looks harder to use | Concerns that the modification would make it harder to collect the samples |
| Too complicated and easy to lose parts | Concerns that the modification makes things overly complicated and that there are more parts to lose. |
| The modification is unnecessary/won't affect participation | Comments regarding the modification being unnecessary or not being related to their FOBT participation (or non-participation) |
| **Perforated collection tool** | |
| Concerns about added costs/waste | Concerns that the modification makes adds to costs and/or waste |
| It would be easier to use | Comments suggesting that the modification would make the sampling procedure easier |
| It would be harder to use/parts might break or get lost | Concerns that the modification would make it easier for things to break and/or get lost |
| More sanitary collection | Comments suggesting that the modification would make the sampling procedure more sanitary |
| It makes it too complicated | Concerns that the modification would make things overly complicated |
| The modification is unnecessary/won't affect participation | Comments regarding the modification being unnecessary or not being related to their FOBT participation (or non-participation) |
| **Ziplock bag** | |
| It would have better concealment for storage | Comments suggesting that the modification would allow for a better seal for the storage of the FOBT sample |
| The modification is unnecessary/won't affect participation | Comments regarding the modification being unnecessary or not being related to their FOBT participation (or non-participation) |
